# Social inequalities in healthcare utilization during Ecuadorian healthcare reform (2007–2017): a before-and-after cross-sectional study

**DOI:** 10.1186/s12889-022-12884-9

**Published:** 2022-03-14

**Authors:** Edy Quizhpe, Enrique Teran, Anni-Maria Pulkki-Brännström, Miguel San Sebastián

**Affiliations:** 1 Department of Epidemiology and Global Health, Umeå University, Umeå, Sweden; 2 Universidad San Francisco de Quito USFQ, Colegio de Ciencias de la Salud, Quito, Ecuador

**Keywords:** Universal health coverage, Socioeconomic inequalities, Unmet healthcare needs, Reform, Latin America, Ecuador

## Abstract

**Background:**

Limited research is available about the impact of healthcare reforms on healthcare utilization according to socioeconomic group. Although most health reforms in Latin America have focused on reducing the gap between the most advantaged and disadvantaged groups and improving the quality of health services, the available information has shown limited progress. Therefore, this study assessed whether the recent Ecuadorian healthcare reform (2007–2017) contributed to decreasing the socioeconomic inequalities in healthcare utilization.

**Methods:**

We used data from the National Living Standards Measurement surveys conducted in 2006 and 2014. Unmet healthcare needs (UHCN) were used as the dependent variable and proxy for difficulties in accessing health services. Place of residence, ethnicity, education and wealth were selected as indicators of socioeconomic status. The slope and relative inequality indexes were calculated for adult men and women for each period and socioeconomic variable. A multiplicative interaction term between midpoint scores and time was applied to estimate changes in inequalities over time. Sample weights were applied to all analyses, and 95% confidence intervals were calculated to assess statistical significance in the regression analysis.

**Results:**

In 2006, the poor, Indigenous, those living in rural areas and with low education had lower access to health services. In 2014, the overall prevalence of UHCN decreased from 27 to 18% and was higher in women than men. Statistically significant reductions of refraining were observed in absolute and relative terms in all social groups, both in men and women.

**Conclusions:**

Our results showed remarkable and significant decreases in inequalities in all examined socioeconomic groups in absolute and relative terms in this period. Although a new model of healthcare was established to achieve universal health coverage, its performance must be continuously evaluated and monitored with specific indicators. Further studies are also needed to identify the main barriers that contribute to UHCN among socially disadvantaged groups.

**Supplementary Information:**

The online version contains supplementary material available at 10.1186/s12889-022-12884-9.

## Background

Over the last three decades, several countries in Latin America have implemented health reforms focused on the privatization of service delivery and decentralization of decision-making processes [1, 2]. However, in opposition to this trend, Ecuador implemented comprehensive and ambitious social reforms from 2007–2017 to become a more inclusive society. Thus, from 2007 to 2014, the poverty headcount ratio and the income inequality (Gini index) were reduced, from 36.7% to 21.5%, and from 53.4 to 44.7, respectively [3]. Besides these, a new healthcare reform aiming to increase access to and quality of healthcare services with a solid base on Alma Ata's primary healthcare principles was introduced [4].

The Ministry of Health (MoH) implemented several mechanisms and health policies as national rector and primary healthcare providers to advance Ecuador towards universal health coverage (UHC). First, the MoH created a new healthcare organization model, the Model of Comprehensive Healthcare (MAIS, *Modelo de Atención Integral de Salud* in Spanish), which focused on promotion and prevention strategies at the community level; primary healthcare teams were introduced to provide more home visits, especially in remote areas with new sanitary infrastructure built across the country. Second, public healthcare services became free of charge at all levels, with a consequent increase in out- and in-patient consultations. Third, the investment in health increased in this period. The public spending on health, as a percentage of GDP, changed from 1.1% in 2007 to 4.2% in 2018 and the health expenditure per capita (US$) increased more than double in the same period (from 209.521 to 516.248). [3, 5]. Finally, though the health reform tried to integrate the health subsystems (MoH, Social Security and the private sector) through several coordination mechanisms, this was not possible to achieve, and the fragmentation of the health system remains [6, 7].

In the Latin American region, almost all countries have made progress in achieving UHC over time in the past decades [8, 9]. However, there is no clear evidence for how these healthcare reforms have reduced social inequalities in access to healthcare. Many countries have reported vast differences in access between social groups, especially in antenatal and maternal services [10]. Also, several studies have shown that women often use more health care services than men [11, 12]. Moreover, most of the available research related to inequalities in healthcare utilization has focused on differences in use by household income, without considering other socially relevant categorizations, thus limiting the broader picture of social inequalities in healthcare [2, 13].

Research on social inequalities in health and healthcare in Ecuador is limited, and studies assessing the role of healthcare reform in reducing those social inequalities are limited [14, 15]. Nevertheless, a recent study assessing the impact of the reform in access to healthcare by household income found a decrease in the gap between poor and rich in use of health services utilization during 2006–2014 [16]. However, the impact of the reform on other relevant social categories has not yet been explored. Therefore, this study assessed whether the recent healthcare reform in Ecuador contributed to decreasing socioeconomic inequalities in healthcare utilization during the period 2006–2014.

## Methods

### Study design and sample

This paper presents a cross-sectional study based on a secondary analysis of the “Living standards measurement survey” conducted in 2006 and 2014 by the National Institute of Statistics and Census (INEC, *Instituto Nacional de Estadísticas y Censos* in Spanish) in Ecuador. The sample selection was probabilistic, stratified and proportional to the population size. Respondents older than 20 were included in the present study, corresponding to 14,639 men and 15,815 women in 2006, and 30,115 men and 32,369 women in 2014. A series of similar questions regarding socioeconomic characteristics, self-reported health and healthcare utilization were included in both surveys. The surveys were applied for one year and conducted in Spanish. To reduce potential bias, several actions were implemented; for instance, the local interviewer properly trained made as many visits as necessary to obtain direct information from the people face-to-face in the selected households [17]. Overall, the proportion of missing data was low (less than 1% in the wealth index) or non-existent in the rest of the social variables and outcomes. Therefore, no measure to deal with missing data was applied.

### Variables

Unmet healthcare needs (UHCN) were used as the healthcare utilization outcome. UHCN, referred to as refraining, is commonly defined as failure to seek healthcare despite an individual’s needs [13, 18]. In addition, self-reported UHCN is widely used as an indicator to evaluate barriers to healthcare utilization [19, 20]. To capture UHCN from the survey, three questions were selected and combined. First, respondents were asked, "In the last month, did you have any illness or injury?" Those who answered affirmative were asked, “Due to that illness or injury, did you visit a doctor, nurse, or traditional healer?” Those who answered no to this second question were categorized as participants with UHCN, except if the reason for refraining was that the illness or injury was mild. This was captured by the answer option “mild illness” to the third question “Why didn’t you visit a doctor, nurse or traditional healer?”.

Residence, ethnicity, education, household wealth, sex and age were used as independent variables that could potentially affect UHCN. Place of residence was defined as living in either an urban or rural area, where parishes with less than 5,000 inhabitants were considered rural. Ethnicity was based on self-identification and divided into two groups: non-Indigenous (including white, mestizos, afro-Ecuadorians, and Montubios) and Indigenous people. Educational level was grouped into four categories: incomplete primary (including illiterate, literate but no formal education and initial education categories); primary; secondary (middle secondary and technical); and higher education (undergraduate and postgraduate). Finally, a household wealth index was calculated based on the household infrastructure and assets (household entrance paving; roof, wall, and floor material; type of house; cooking facilities; cooking fuel; type of toilet; water source; lighting source; landline telephone; home internet; satellite TV; and household waste disposal) using principal component analysis (PCA). The index was generated from a total of 15 categorical variables. Then, factor scores were computed, principal components were extracted, and the first was used to measure economic status [21]. The obtained index was divided into quintiles, the first one representing the richest.

### Statistical analysis

Population characteristics were first summarized with descriptive statistics disaggregated by sex and year. The UHCN by each socioeconomic variable was also stratified by sex and year. To examine the absolute and relative socioeconomic inequalities in UHCN, the slope index of inequality (SII) and the relative index of inequality (RII) were calculated. The advantage of these measures resides in their capacity to summarize the health inequality in one number, which makes comparisons over time more interpretable and weight for the population size in the different social categories [22, 23]. This study used the most socially advantaged category (urban, non-Indigenous, highly educated and high wealth index) as the reference group. A log-binomial regression model was applied with the log link function for calculating the RII and the identity link function for SII between the health outcome and the predictors variables. Ridit scores, corresponding to the average cumulative proportion of the population in each socioeconomic indicator category, were first created and used to estimate the SII and RII in men and women separately for each socioeconomic variable adjusted by age in each of the analysed periods (2006 and 2014). Values of 0 in the case of SII and 1 in RII indicate no inequalities. Conversely, values above 0 and 1 in SII and RII indicate a higher UHCN among the most socially disadvantaged compared to the least socially disadvantaged groups. After merging the two national surveys, a multiplicative interaction term between ridit and time was included in the regression model to estimate changes in the inequalities over time. Sample weights were applied to all analyses, and 95% confidence intervals (95% CI) were calculated to assess statistical significance in the regression analysis. All analyses were conducted using the Stata 15.1 statistical software. Finally, the study protocol was performed following the relevant guidelines.

## Results

Table 1 shows the main socio-demographic characteristics of the study population during the two periods. A similar age and residence distribution was found in both periods. About 50% of those interviewed belonged to the age group 20 to 39 years, 70% of participants lived in an urban area and the proportion of Indigenous people was around 7%. The proportion with secondary or higher education increased among both men and women over the study period.

**Table 1:** Socioeconomic characteristics of participants stratified by sex 2006 and 2014, Ecuador (weighted samples)

	**2006**		**2014**	
**Variable**	**Men**	**Women**	**Men**	**Women**
**Categories**	***N (%)***	***N (%)***	***N (%)***	***N (%)***
**Age**				
20 – 39	7636 (52.16)	8225 (52.01)	14,744 (48.96)	15,803 (48.82)
40 -64	5394 (36.85)	5750 (36.36)	11,537 (38.31)	12,389 (38.27)
More than 64	1609 (10.99)	1840 (11.64)	3834 (12.73)	4177 (12.91)
**Residence**				
Urban	9888 (67.54)	11,084 (70.09)	21,537 (71.51)	23,691 (73.19)
Rural	4751 (32.46)	4731 (29.91)	8578 (28.49)	8678 (26.81)
**Ethnicity**				
Non-indigenous	13,612 (92.98)	14,767 (93.37)	28,053 (93.15)	30,142 (93.12)
Indigenous	1027 (7.02)	1048 (6.63)	2062 (6.85)	2227 (6.88)
**Level of education**				
Higher (highest)	2931 (20.02)	3124 (19.76)	6439 (21.38)	7033 (21.73)
Secondary	4622 (31.57)	4876 (30.83)	10,710 (35.57)	11,164 (34.49)
Primary	6193 (42.31)	6272 (39.66)	10,867 (36.08)	11,075 (34.22)
Incomplete primary (lowest)	892 (6.09)	1542 (9.75)	2098 (6.97)	3096 (9.57)
**Household wealth index**				
1st quintile (highest)	3525 (24.55)	4216 (26.84)	9168 (31.06)	10,448 (32.46)
2nd quintile	3241 (22.57)	3707 (23.60)	6815 (23.09)	7672 (23.83)
3rd quintile	2917 (20.31)	3145 (20.02)	5632 (19.08)	5938 (18.45)
4th quintile	2422 (16.87)	2478 (15.78)	4176 (14.15)	4500 (13.98)
5th quintile (lowest)	2254 (15.70)	2161 (13.76)	3726 (12.62)	3633 (11.29)

In 2006, 27% of participants reported refraining from seeking healthcare despite a perceived need, the proportion being higher in women (28.95%) than in men (24.33%). The prevalence of UHCN was higher in disadvantaged groups – that is, among those living in rural areas, being Indigenous, with low education and being poor. In 2014, the overall prevalence of refraining from seeking healthcare decreased to 18%, slightly higher in women (19.25%) than in men (17.32%). Reductions in prevalence were observed in all socioeconomic groups (Table 1S in Appendix) and these differences between periods were statistically significant (*p* < 0.01).

### Trends in socioeconomic inequalities

Tables 2 and 3 display the SII and RII for UHCN in 2006 and 2014 for men and women, respectively. Overall, we observed significant reductions in socioeconomic inequalities over time in absolute and relative terms.

**Table 2:** Absolute and relative index of inequality in unmet health care needs in men 2006–2014, Ecuador

	**2006** **Estimate (95% CI)**^**a**^		**2014** **Estimate (95% CI)**^**a**^		**Absolute and Relative differences** **2006-2014**^**a**^	
**Residence**						
SII	12.68	(9.65–15.71)	5.38	(3.47–7.28)	-7.46	(-10.79,-4.12)
RII	1.68	(1.49–1.88)	1.36	(1.22–1.51)	0.82	(0.71–0.96)
**Ethnicity**						
SII	14.46	(8.67–20.25)	4.62	(1.17–8.07)	-9.94	(-16.21,-3.68)
RII	1.69	(1.40–2.04)	1.32	(1.10–1.58)	0.78	(0.61–1.01)
**Education**						
SII	22.16	(19.51–24.81)	11.48	(9.87–13.09)	-10.21	(-12.98,-7.45)
RII	2.46	(2.19–2.77)	1.90	(1.73–2.09)	0.85	(0.74–0.97)
**Household wealth**						
SII	20.84	(18.36–23.32)	13.50	(11.90–15.09)	-7.48	(-10.23,-4.72)
RII	2.22	(2.01–2.46)	1.99	(1.83–2.17)	0.90	(0.79–1.03)

^a^age-adjusted

*CI* Confidence interval

*SII* Slope index of inequality

*RII* Relative index of inequality

**Table 3:** Absolute and relative index of inequality in unmet health care needs in women 2006–2014, Ecuador

	**2006** **Estimate (95% CI)**^**a**^		**2014** **Estimate (95% CI)**^**a**^		**Absolute and Relative differences** **2006-2014**^**a**^	
**Residence**						
SII	14.34	(11.20–17.48)	2.75	(0.83–4.66)	-11.43	(-14.86,-8.00)
RII	1.63	(1.47–1.80)	1.18	(1.07–1.30)	0.74	(0.64–0.85)
**Ethnicity**						
SII	21.19	(15.15–27.23)	4.32	(0.90–7.75)	-16.76	(-23.20,-10.33)
RII	1.94	(1.66–2.26)	1.22	(1.03–1.43)	0.63	(0.51–0.79)
**Education**						
SII	23.77	(21.10–26.43)	8.96	(7.36–10.55)	-12.12	(-14.83,-9.42)
RII	2.29	(2.08–2.52)	1.60	(1.47–1.74)	0.86	(0.76–0.96)
**Household wealth**						
SII	25.68	(23.13–28.24)	10.21	(8.60–11.81)	-15.39	(-18.21,-12.56)
RII	2.27	(2.08–2.47)	1.59	(1.47–1.72)	0.71	(0.63–0.80)

^a^age-adjusted

*CI* Confidence interval

*SII* Slope index of inequality

*RII* Relative index of inequality

In detail, among men, all disadvantaged social groups had a statistically significantly higher prevalence (absolute and relative) of UHCN than the better off in both years. Statistically significant reductions in absolute socioeconomic inequalities (SII) were observed in all socioeconomic variables over time. The magnitude of the reductions was significantly large and up to a decrease of over 10 percentage points in the case of education (95% CI: − 12.98, − 7.45). Ethnicity (SII: 4.62; 95% CI: 1.17–8.07) and residence (SII: 5.38; 95% CI: 3.47–7.28) showed the lowest values of inequalities in 2014. Similarly, the inequalities in relative terms decreased in all socioeconomic groups. For instance, the RII in education moved from 2.46 in 2006 to 1.90 in 2014, the difference being statistically significant (RII = 0.85; 95% CI: 0.74, 0.97).

Among women, and similar to men, all disadvantaged social groups had a statistically significantly higher prevalence (absolute and relative) of UHCN than the better off in both years. Considerable and significant reductions in absolute differences over time were observed in all social groups, with declines over 12 percentage points, especially in the case of ethnicity (SII difference: − 16.76; 95% CI: − 23.20, − 10.33), wealth (SII difference: − 15.39; 95% CI − 18.21, − 12.56) and education (SII difference: − 12.12; 95% CI: − 14.83, − 9.42). A similar pattern was observed for the relative measurements, with reductions in all socioeconomic variables over the two periods. A striking 37% difference decrease was observed for ethnicity (RII = 0.63; 95% CI: 0.51, 0.79) with important statistically significant reductions for wealth (RII = 0.71; 95% CI: 0.63, 0.80), residence (RII = 0.74; 95% CI: 0.64, 0.85) and education (RII = 0.86; 95% CI: 0.76, 0.96).

## Discussion

The present study illustrates the effects of Ecuadorian healthcare reform (2007–2017) on socioeconomic inequalities in healthcare utilization in men and women. Our results showed that refraining from healthcare use was reduced from 27 to 18% between 2006 and 2014, with remarkable decreases in all socioeconomic groups in both, absolute and relative terms over time. The prevalence of refraining of 18% in Ecuador in 2014 was lower compared to the situation in Colombia and Paraguay (around 25%) and much lower than in Perú (65.9%), in 2016 [24].

In all socioeconomic groups, women had the highest proportion of refraining from seeking healthcare compared to men. Some studies have shown that women may delay care-seeking due to embarrassment and intimidation by healthcare providers. Likewise, Indigenous and poor women have been reported to be less likely to recognize health risks and seek care due to the lack of integrative socio-cultural practice in delivering health services [25, 26].

The observed significant reductions of UHCN, mainly among women and in all disadvantaged socioeconomic groups, could to some extent be explained by some of the arguments provided by De Paepe et al. [27]. First, the Ecuadorian government made a substantial financial investment. Public investment as total health expenditure increased from 1.08% of the national gross domestic product (GDP) in 2006 to 2.92% in 2016 [28]. These resources were invested in drug supplies, ambulances, hospital equipment, specialized mobile units and healthcare workers. Overall, there is strong evidence that the government's appropriate national and subnational investments can lead to better health outcomes if effectively used [29, 30].

The MoH also implemented other mechanisms to stimulate the use of healthcare services, such as a conditional cash transfer scheme to promote child and maternal health services. However, its impact on socially vulnerable women has been debatable [31]. Likewise, cost-recovery mechanisms were established through the Integral Health Public Network (RPIS, *Red Publica Integral de Salud* in Spanish) and Complementary Network (*Red Complementaria* in Spanish) between public–private partnerships to support the demand for healthcare utilization and address the current fragmentation of the health system. However, reports have pointed that these partnerships mainly contributed to an increased accumulation of capital in the private sector, especially in pharmaceutical and insurance industries, and hindered the reduction of social inequalities in healthcare [32].

Second, all MoH health services became progressively free of charge for all citizens. Consequently, the number of consultations at all levels of healthcare increased from 16 to 42 million between 2006 and 2015 [33]. According to UHC principles, expanding services to difficult-to-reach areas can also decrease socioeconomic inequalities in healthcare. An increase in coverage was attained through community health teams called the Healthcare Basic Team (EBAS, *Equipo Básico de Atención de Salud* in Spanish). More than 4,000 physicians were contracted for the EBAS program. Routine home visits were performed by these teams offering preventive and curative services, particularly in deprived and remote areas.

A third argument relates to the comprehensive primary healthcare model implemented under the Ecuadorian health reform [34]. It has been argued that models of health systems based on Alma Ata’s principles, as in the case of Paraguay and El Salvador, can better decrease socioeconomic inequalities in healthcare use compared to health insurance–based models, such as those used in Peru and Colombia [24].

Although access to care among most disadvantaged socioeconomic groups improved over time, the present study found that considerable educational and wealth inequalities persisted. Despite the reform and government-defined priority and vulnerable groups based on needs, an inadequate health workforce and infrastructure distribution may have limited care in more impoverished and remote areas. The synergism between barriers such as distance and travel costs could discourage these groups from accessing the healthcare system. Studies have also reported that free public services are often perceived as having low quality, even more so among the poor than the rich [35, 36]. Such perceptions may have limited the impact of removing user fees on inequalities in UHCN.

### Methodological considerations

This study has strengths and limitations that should be considered when interpreting the results. One main strength is the national representation of the different socioeconomic groups before and during the reform implementation. In addition, the use of two complex measures of health inequalities, both in absolute and relative terms, contributes to providing a broader picture of the impact on social inequalities in healthcare utilization and to assessing the evolution over time. However, the questions in the survey regarding UHCN were related to healthcare utilization for curative but not preventive needs. Besides, one month is possibly a short time to evaluate UHCN, which means that the magnitude of the total utilization could only be partially evaluated. Also, the question in the surveys did not allow separate analysis between formal medical care and traditional healers. In addition, response and recall bias could have been present for some questions due to the nature of the study design, despite the thorough training of the interviewers.

Further, we are aware that the before-after cross-sectional study design does not allow to infer a causal attribution to the reform since other factors could have also influenced the UHCN. However, this was the only possible design to be applied given the available data in the country. Finally, although the health reform was implemented during 2007–2017, the study included only two points in 2006 and 2014, which limits the assessment of trends. Because the reform continued until 2017, it would have been interesting to observe if the reform results were different, but no data were yet available.

## Conclusions

This study sought to assess whether the recent healthcare reform contributed to reducing socioeconomic inequalities in access to healthcare services in Ecuador. Our results showed that refraining from healthcare use was reduced from 27 to 18% from 2006 to 2014, with significant decreases in all examined socioeconomic groups. Ecuador thus provides a relevant example of a healthcare model aiming to achieve UHC. However, its performance must be continuously evaluated and monitored with specific indicators. Further studies are also needed to identify the main barriers to refraining from healthcare access among socially disadvantaged groups. Finally, national social policies must continue to address the social determinants of healthcare, and new healthcare reforms should deal with the fragmentation of the existing healthcare system to achieve UHC.

## Supplementary Information

**Additional file 1:** **Table 1S.** Socioeconomic prevalence of unmet health care needs stratifying by sex in 2006 and 2014, Ecuador (weighted samples).

## Data Availability

Data used in this study are publicly available and can be retrieved from https://www.ecuadorencifras.gob.ec/institucional/home/.

## Abbreviations

UHCN: Unmet healthcare needs
MoH: Ministry of Health
UHC: Universal Health Coverage
MAIS: Modelo de Atención Integral de Salud
INEC: Instituto Nacional de Estadísticas y Censos
SII: Slope Index of Inequality
RII: Relative Index of Inequality
GDP: National Gross Domestic Product
RPIS: Red Publica Integral de Salud
EBAS: Equipo Básico de Atención de Salud
