# Supplementary material for: Social inequalities in healthcare utilization during Ecuadorian healthcare reform (2007–2017): a before-and-after cross-sectional study
Source: BMC Public Health. 2022 Mar 14;22:499. doi: 10.1186/s12889-022-12884-9 (PMC8922889; doi:10.1186/s12889-022-12884-9)
Supplement: Supplementary file 1 — Additional file 1: Table 1S. Socioeconomic prevalence of unmet health care needs stratifying by sex in 2006 and 2014, Ecuador (weighted samples). [file 12889_2022_12884_MOESM1_ESM.docx]

**Appendix. Table 1S.** **Socioeconomic prevalence of unmet health care needs stratifying by sex in 2006 and 2014, Ecuador (weighted samples)**

|  | **Men** |  | **Women** |  |
| --- | --- | --- | --- | --- |
| **Variable** | **2006**  **N (%)** | **2014**  **N (%)** | **2006**  **N (%)** | **2014**  **N (%)** |
| **Total *** | 3562 (24.33) | 5214 (17.32) | 4578 (28.95) | 6231 (19.25) |
| **Residence*** |  |  |  |  |
| Urban | 2170 (21.95) | 3519 (16.34) | 2950 (26.61) | 4422 (18.67) |
| Rural | 1391 (29.29) | 1694 (19.76) | 1628 (34.41) | 1809 (20.85) |
| **Ethnicity** |  |  |  |  |
| Mestizos/afro/white | 3240 (23.80) | 4806 (22.78) | 4166 (28.22) | 5753 (19.09) |
| Indigenous | 322 (31.33) | 408 (24.06) | 411 (39.23) | 478 (21.46) |
| **Education *** |  |  |  |  |
| Higher (highest) | 417 (14.25) | 767 (11.92) | 568 (18.21) | 946 (13.46) |
| Secondary | 976 (21.13) | 1613 (15.06) | 1216 (24.94) | 1890 (16.93) |
| Primary | 1826 (29.49) | 2331 (21.46) | 2119 (33.78) | 2639 (23.83) |
| Incomplete primary (lowest) | 340 (38.22) | 501 (23.91) | 673 (43.68) | 754 (24.38) |
| **Household wealth*** |  |  |  |  |
| 1st quintile (highest) | 554 (15.72) | 1152 (12.57) | 804 (19.09) | 1575 (15.08) |
| 2nd quintile | 714 (22.05) | 1050 (15.42) | 1003 (27.07) | 1545 (20.14) |
| 3rd quintile | 784 (26.90) | 1185 (21.04) | 1031 (32.79) | 1276 (21.49) |
| 4th quintile | 702 (28.98) | 892 (21.36) | 866 (34.99) | 991 (22.04) |
| 5th quintile (lowest) | 723 (32.11) | 800 (21.48) | 845 (39.10) | 804 (22.14) |

* *P* < 0.01 Chi square test was performed between sex and period.
